# Successive onset of Vogt-Koyanagi-Harada syndrome in father and son

**DOI:** 10.1186/s12886-023-02851-2

**Published:** 2023-03-21

**Authors:** Hougang Li, Shuo Sun, Yanrui Zhang, Jinfeng Liu, Xuzheng Zhao, Guixia Zhao

**Affiliations:** 1 Tangshan Institute of Ophthalmology, Tangshan Eye Hospital, 063000 Hebei, China; 2 North China University of Science and Technology Affiliated Eye Hospital, 063000 Hebei, China; 3 Tianjin Key Laboratory of Retinal Functions and Diseases, Tianjin Branch of National Clinical Research Center for Ocular Disease, Eye Institute, Tianjin Medical University Eye Hospital, 300384 Tianjin, China

**Keywords:** Vogt‒Koyanagi‒Harada syndrome, Familial cases, Genetic factors

## Abstract

**Background:**

Vogt‒Koyanagi‒Harada (VKH) disease is a multifactorial systemic autoimmune disorder against melanocytes that is characterized by panuveitis. Familial occurrence of VKH disease is rare. Here, we report two cases of a father and his son with characteristic manifestations of VKH disease.

**Case presentation:**

A 53-year-old male with typical clinical symptoms of VKH disease was referred to Tangshan Eye Hospital. Examination showed the presence of ciliochoroidal effusion and exudative retinal detachment in both eyes. The patient was given intravenous methylprednisolone 120 mg for 2 days and intravenous methylprednisolone 80 mg for 1 day followed by 48 mg (1 mg/kg/day) oral methylprednisolone daily, accompanied by oral azathioprine 50 mg daily. Cycloplegic agent (0.5% tropicamide three times daily [TID]) was added. The patient was free of symptoms and recurrence within more than 1-year-follow-up period, the best corrected visual acuity (BVCA) was increased and maintained in both eyes with complete resolution of subretinal fluid. One year and nine months later, case 2 (his son) also presented with the typical clinical symptoms of VKH disease at 29 years of age. The son also recovered from VKH disease after routine and standard treatment.

**Conclusions:**

To the best of our knowledge, this is the first VKH disease case report of a father-son relationship. Although genetic factors have been demonstrated to be involved in the pathogenesis of VKH disease, the different inheritance modes of VKH patients need to be further explored and studied.

## Background

Vogt–Koyanagi–Harada (VKH) disease is a rare bilateral granulomatous uveitis involving pigmented structures [1]. Although the precise etiology of VKH disease is still largely unknown, accumulating previous studies have suggested that genetic factors play an essential role in VKH disease development [2, 3]. Familial occurrence of VKH disease is rare, while authors still provide some evidence to suggest that the condition may be inherited [4–6]. Here, two cases of a father and his son with characteristic manifestations of VKH disease are presented.

### Case presentation

#### Case 1

A 53-year-old male with eye pain and blurred vision in both eyes, accompanied by severe headaches, nausea, and vomiting for 2 days, presented to a local eye clinic. He was diagnosed with bilateral primary angle closure glaucoma and was treated with topical pilocarpine combined with intravenous mannitol administration. However, there was no improvement in the symptoms after treatment. Thus, he was referred to our hospital.

**Fig. 1:**
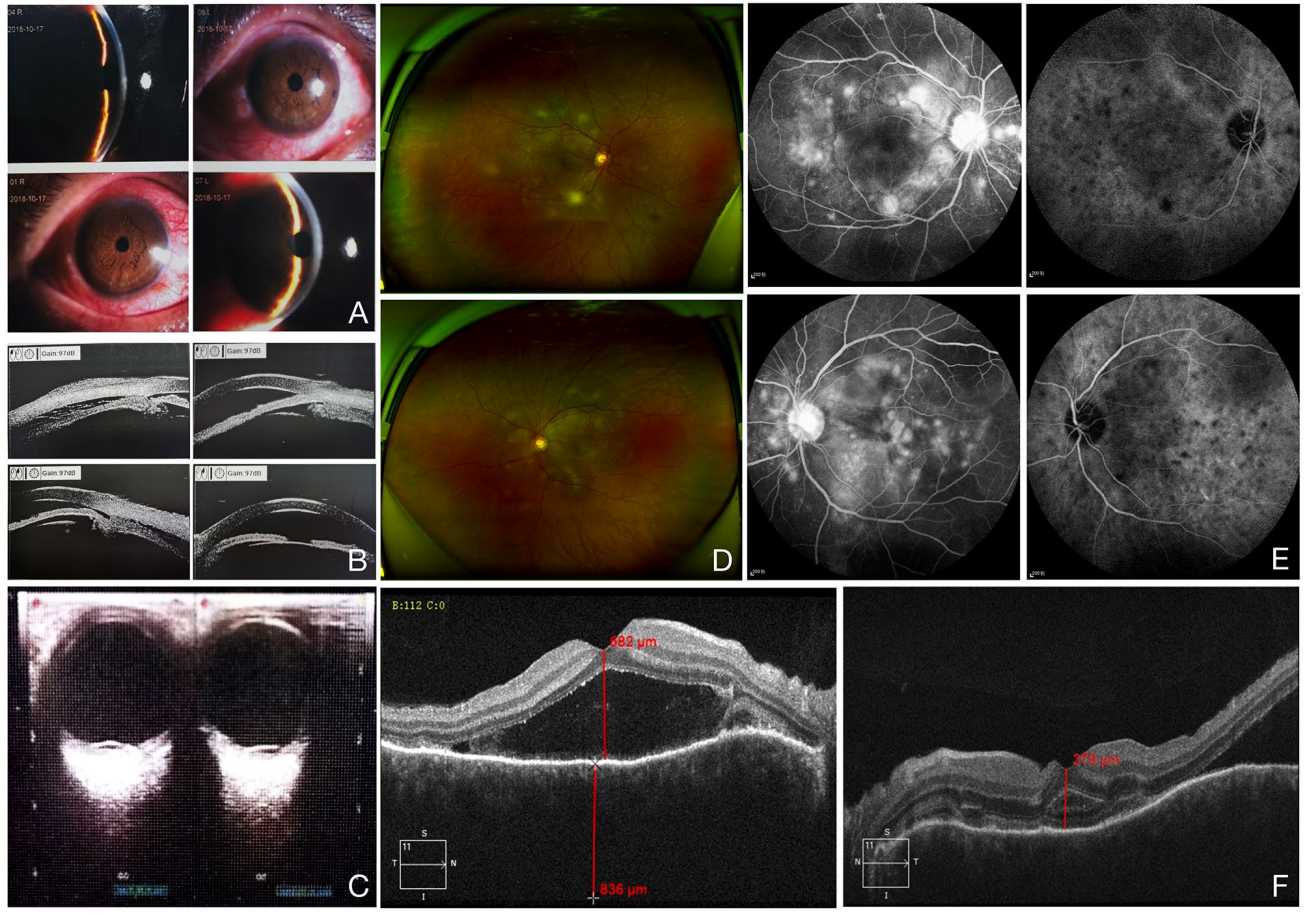
Representative images of case 1. **(A).** The anterior chambers were shallow, and the conjunctivas were congested with mild corneal edema. **(B).** UBM showing the presence of ciliochoroidal effusion and ciliary body detachment. **(C).** B-scan ultrasonography showing retinal detachment with ciliochoroidal detachment. **(D).** Dilated fundus examination showing multifocal choroiditis lesions, multifocal SRDs and tortuous retinal vessels in both eyes. **(E).** FFA images show bilateral multiple lake hyperfluorescence, pooling within the subretinal space in the late phase, and ICGA images show multiple hypofluorescent dark dots. **(F).** OCT showing exudative retinal detachment in both eyes.

On initial presentation, ophthalmic examination showed best corrected visual acuity (BVCA) of 20/100 in the right eye and 20/70 in the left eye and intraocular pressure (IOP) of 45 and 41 mmHg. He denied any family history or previous history of ocular diseases. His past medical history included hypertension and kidney stones. The conjunctiva was congested with mild corneal edema. The anterior chambers were shallow and flare, and cells could not be examined in detail in both eyes (see Fig. 1A). The pupillary light reflexes were absent due to the use of pilocarpine. Mild lens opacities were seen. The posterior segment could not be detected in detail. UBM showed the presence of ciliochoroidal effusion and ciliary body detachment in both eyes (see Fig. 1B). B-scan ultrasonography indicated retinal detachment with ciliochoroidal detachment in both eyes (see Fig. 1C). Optical coherence tomography (OCT) also demonstrated exudative retinal detachment in both eyes (see Fig. 1F). The increased IOP did not respond to anti-glaucoma therapy. Uveitis systemic workup was negative. After consultation, the patient was initially diagnosed with bilateral incomplete VKH disease with acute angle-closure glaucoma according to the revised diagnostic criteria for VKH. The patient was given intravenous methylprednisolone 120 mg for 2 days and intravenous methylprednisolone 80 mg for 1 day followed by 48 mg (1 mg/kg/day) oral methylprednisolone daily, accompanied by oral azathioprine 50 mg daily. Cycloplegic agent (0.5% tropicamide three times daily [TID]) was added. Dilated fundus examination revealed multifocal choroiditis lesions, multifocal serous retinal detachments (SRDs) and tortuous retinal vessels in both eyes (see Fig. 1D). Fundus fluorescein angiography (FFA) revealed bilateral multiple lake hyperfluorescence, pooling within the subretinal space in the late phase. In indocyanine green angiography (ICGA), multiple hypo-fluorescent dark dots were detected and remained in the late phase (see Fig. 1E). The patient was discharged from our hospital and continued with the same prescription. He was asked for a monthly follow-up visit.

The patient was free of symptoms and recurrence within more than 1-year-follow-up period, the BVCA was increased and maintained at 20/25 in his right eye and 20/20 in his left eye. With the complete resolution of subretinal fluid, we observed mild sunset glow fundus and chorioretinal depigmentation in both eyes (see Fig. 3A, C and D). Moreover, the macular appearance of both eyes almost returned to normal with slightly increased choroidal thickness.

### Case 2

**Fig. 2:**
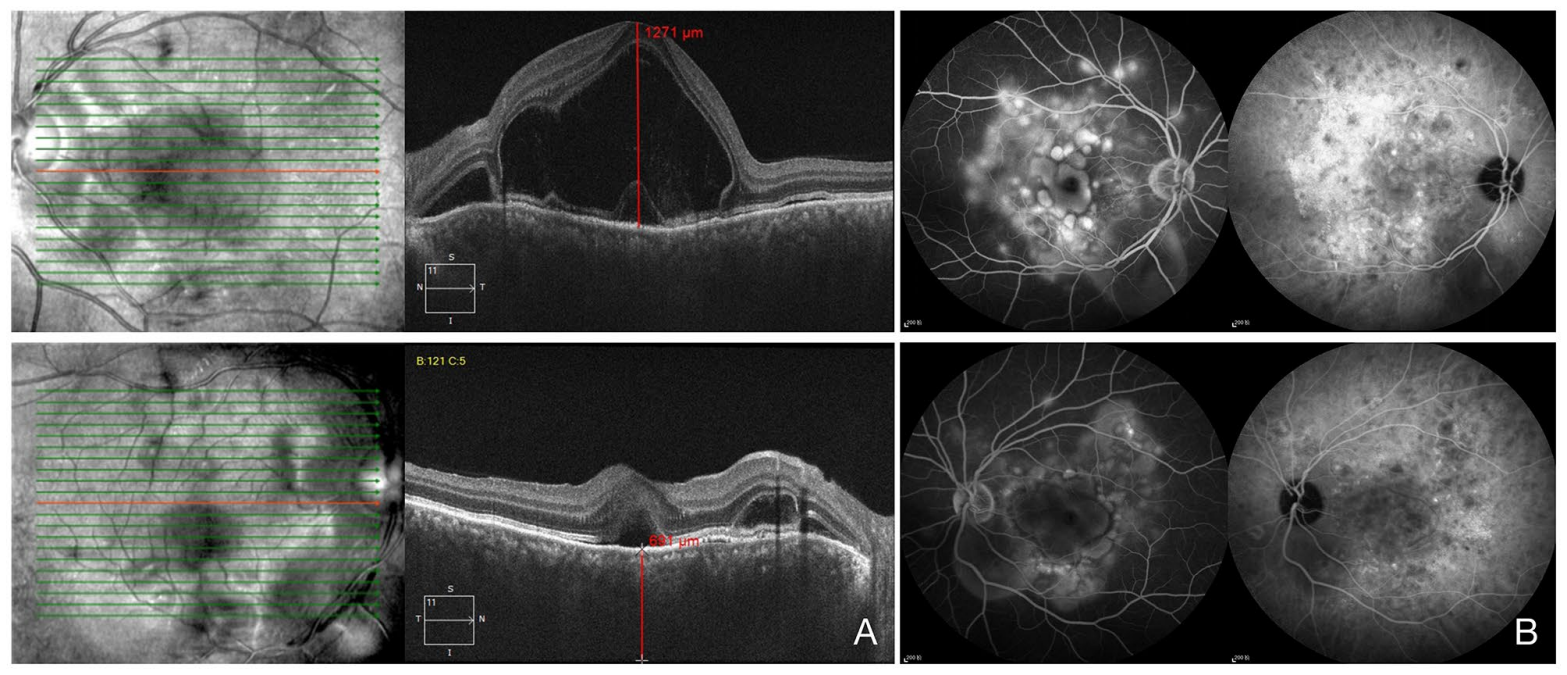
Representative images of case 2. **(A).** OCT showing exudative retinal detachment in both eyes. **(B).** FFA images show bilateral multiple lake hyperfluorescence, pooling within the subretinal space in the late phase, and ICGA images show multiple hypofluorescent dark dots.

Two years after the onset of case 1, a 29-year-old male, who is case 1’s son, presented with blurred vision in both eyes without headaches, nausea and vomiting for 3 days. BCVA of 20/70 in the right eye and 20/500 in the left eye with normal IOP. He also denied any past ocular, medical or family history. The conjunctiva was white and quiet. Dash-like keratic precipitates (KP) were observed, and flare (+) and cells (+) were present in the anterior chamber in both eyes. The pupillary light reflexes were sluggish with vitreous opacity. Posterior segment examination showed multiple serous retinal detachments and retinal folds. OCT also demonstrated exudative retinal detachment with macular edema and obviously increased choroidal thickness in both eyes (see Fig. 2A). The features of FFA/ICGA were similar to those of his father (see Fig. 2B). Uveitis systemic workup was negative. The laboratory data were normal. According to the revised VKH diagnostic criteria, this patient was diagnosed with bilateral incomplete VKH disease. The patient was given retrobulbar dexamethasone 5 mg once and oral prednisolone 60 mg daily, accompanied by 0.5% tropicamide TID.

We planned to taper off prednisolone gradually and asked him for a monthly follow-up visit.

**Fig. 3:**
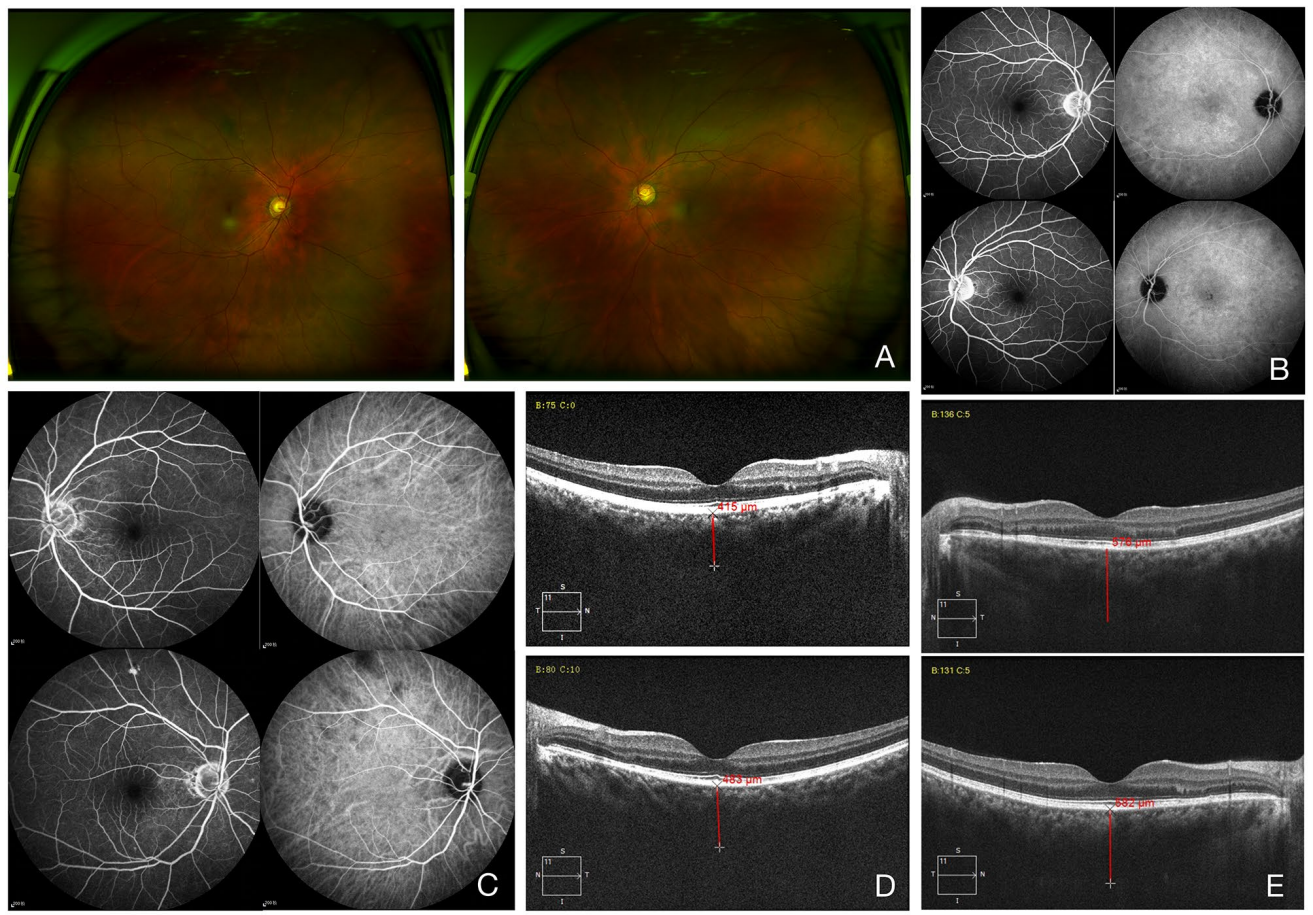
Representative images during patient follow-up. Father (A, C and D) and his son (B and E) were symptom-free after more than 1 year of follow-up

During the following year, the patient was also free of symptoms and recurrence. The BVCA was increased and maintained at 20/20 in both eyes. The fundus almost returned to normal as well as those of his father (see Fig. 3B and E).

### Discussion and conclusions

VKH is an autoimmune disease characterized by granulomatous uveitis in both eyes and is usually accompanied by meningeal irritation, auditory dysfunction, and skin and hair abnormalities [7]. VKH syndrome more frequently affects people of color, especially Asians, Middle Easterners, South Americans and American Indians. Recent studies have demonstrated multiple genetic factors that might be related to the pathogenesis of VKH disease [8]. In 1981, Ohno et al. found a strong relationship between human leukocyte antigens (HLAs) and VKH disease [9]. With the continuous deepening of VKH related research, an increasing number of HLA genotypes have been found to be correlated with VKH disease in recent years, and the association of different HLA antigens or alleles with VKH disease depends on race [10, 11]. Furthermore, some general genetic risk factors, such as *interleukin-23 receptor-C1orf141* and *2-aminoethanethiol dioxygenase-zinc finger protein 365-early growth response 2*, were shown to be correlated with Japanese patients with VKH disease [12]. Other immune response genes, including complement factor H, cytotoxic T-lymphocyte antigen 4 and macrophage migration inhibitory factor, are involved in the development of VKH disease, although they are not the main contributors [13]. In our cases, the HLA-typing results of two patients showed both father and son expressed HLA-DRB1*0405 and HLA-DQB1*0401, which could be potential genetic markers for VKH seen in Asian people [10, 14](Table 1). The HLA association suggests an immunogenetic predisposition to VKH in these familial cases.

**Table 1:** HLA-typing results

	Father		Son	
Locus	Allele 1	Allele 2	Allele 1	Allele 2
HLA-A	24:02:01	24:02:01	24:02:01	11:01:01
HLA-B	54:01:01	57:01:01	15:07:01	57:01:01
HLA-C	06:02:01	01:02:01	06:02:01	03:03:01
HLA-DRB1	07:01:01	04:05:01	07:01:01	04:05:01
HLA-DQB1	04:01:01	03:96	04:01:01	03:96
HLA-DRB4	01:03:01	01:03:01	01:03:01	01:03:01
HLA-DQA1	02:01:01	03:03:01	02:01:01	03:03:01
HLA-DPB1	02:01:02	05:01:01	05:01:01	03:01:01
HLA-DPA1	01:03:01	02:02:02	01:03:01	02:02:02

In addition to genetic factors, viral infection and environmental factors can also affect the pathogenesis of VKH disease, which likely involves the T-cell-mediated autoimmune response to melanocyte-specific antigens [15]. Recent studies have suggested that this kind of response and genetic predisposition is triggered by infection-related molecular mimicry [16].

Viral infections and autoimmune disease have long been linked, but there was no clear history of infection in these cases. In recent years, COVID-19 infection and COVID-19 vaccines have also been found as stimulators of VKH disease [17, 18]. Although a causal relationship between COVID-19 and uveitis may exist, both father and son are not vaccinated against COVID. And COVID-19 antigen and antibody test were also negative when they came to our clinic.

VKH syndrome is not a common disease, and the incidence is approximately 20–50/100,000 yearly [19]. It is extremely rare for fathers and sons to suffer from VKH disease within a short time. Therefore, it is worth further studying and discussing whether the connection between these two cases is accidental or inevitable. The HLA-typing results can provide insights on immunogenetic predisposition in VKH disease. Genome-wide association study analysis may help us to screen and identify the new loci associated with VKH syndrome in this father-son relationship. Although twin and sibling studies have provided insights into familial cases with VKH disease and suggested that hereditary factors play an essential role in the pathogenesis of VKH disease [4–6], this case report of the father-son relationship provides us with a new genetic perspective to observe and analyse VKH disease pathogenesis, and the family history in VKH patients needs special attention.

To the best of our knowledge, this is the first VKH disease case report of a father-son relationship. And the different inheritance modes of VKH patients need to be further explored and studied.

## Data Availability

The datasets used and/or analysed during the current study available from the corresponding author on reasonable request.

## List of abbreviations

***BVCA***: *Best Corrected Visual Acuity*
***FFA***: *Fundus fluorescein angiography*
***HLA***: *Human Leukocyte Antigens*
***ICGA***: *Indocyanine green angiography*
***IOP***: *Intraocular pressure*
***OCT***: *Optical coherence tomography*
***SRDs***: *serous retinal detachments*
***TID***: *Three times daily*
***VKH***: *Vogt-Koyanagi-Harada*
